# Advances in Catchment Science, Hydrochemistry, and Aquatic Ecology Enabled by High-Frequency Water Quality Measurements

**DOI:** 10.1021/acs.est.2c07798

**Published:** 2023-03-13

**Authors:** Magdalena Bieroza, Suman Acharya, Jakob Benisch, Rebecca N. ter Borg, Lukas Hallberg, Camilla Negri, Abagael Pruitt, Matthias Pucher, Felipe Saavedra, Kasia Staniszewska, Sofie G. M. van’t Veen, Anna Vincent, Carolin Winter, Nandita B. Basu, Helen P. Jarvie, James W. Kirchner

**Affiliations:** 1 Department of Soil and Environment, SLU, Box 7014, Uppsala 750 07 Sweden; 2 Department of Environment and Genetics, School of Agriculture, Biomedicine and Environment, La Trobe University, Albury/Wodonga Campus, Victoria 3690, Australia; 3 Institute for Urban Water Management, TU Dresden, Bergstrasse 66, Dresden 01068, Germany; 4 Environment Research Centre, Teagasc, Johnstown Castle, Wexford Y35 Y521, Ireland; 5 The James Hutton Institute, Craigiebuckler, Aberdeen AB15 8QH, United Kingdom; 6 School of Archaeology, Geography and Environmental Science, University of Reading, Whiteknights, Reading RG6 6AB, United Kingdom; 7 Department of Biological Sciences, University of Notre Dame, Notre Dame, Indiana 46556, United States; 8 Institute of Hydrobiology and Aquatic Ecosystem Management, Vienna University of Natural Resources and Life Sciences, Gregor Mendel Straße 33, Vienna 1180, Austria; 9 Department for Catchment Hydrology, Helmholtz Centre for Environmental Research - UFZ, Theodor-Lieser-Straße 4, Halle (Saale) 06120, Germany; 10 Department of Earth and Atmospheric Sciences, University of Alberta, Edmonton, Alberta T6G 2E3, Canada; 11 Department of Ecoscience, Aarhus University, Aarhus 8000, Denmark; 12 Envidan A/S, Silkeborg 8600, Denmark; 13 Environmental Hydrological Systems, University of Freiburg, Friedrichstraße 39, Freiburg 79098, Germany; 14 Department of Hydrogeology, Helmholtz Centre for Environmental Research - UFZ, Permoserstr. 15, Leipzig 04318, Germany; 15 Department of Civil and Environmental Engineering and Department of Earth and Environmental Sciences, and Water Institute, University of Waterloo, Waterloo, Ontario N2L 3G1, Canada; 16 Water Institute and Department of Geography and Environmental Management, University of Waterloo, Waterloo, Ontario N2L 3G1, Canada; 17 Department of Environmental System Sciences, ETH Zurich, Zurich CH-8092, Switzerland; 18 Swiss Federal Research Institute WSL, Birmensdorf CH-8903, Switzerland

**Keywords:** Catchment science, stream hydrochemistry, aquatic ecology, high-frequency, water quality monitoring, optical sensors

## Abstract

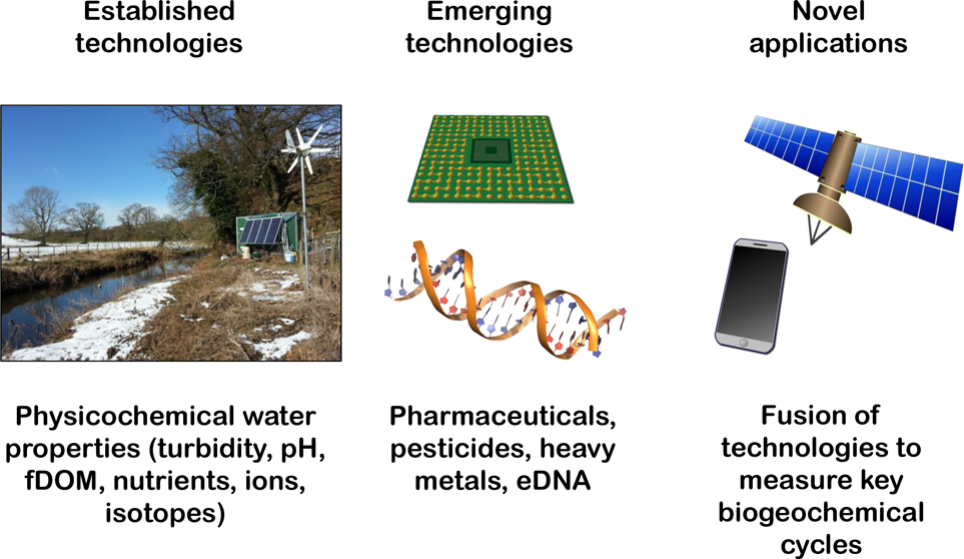

High-frequency water quality measurements in streams and rivers have expanded in scope and sophistication during the last two decades. Existing technology allows *in situ* automated measurements of water quality constituents, including both solutes and particulates, at unprecedented frequencies from seconds to subdaily sampling intervals. This detailed chemical information can be combined with measurements of hydrological and biogeochemical processes, bringing new insights into the sources, transport pathways, and transformation processes of solutes and particulates in complex catchments and along the aquatic continuum. Here, we summarize established and emerging high-frequency water quality technologies, outline key high-frequency hydrochemical data sets, and review scientific advances in key focus areas enabled by the rapid development of high-frequency water quality measurements in streams and rivers. Finally, we discuss future directions and challenges for using high-frequency water quality measurements to bridge scientific and management gaps by promoting a holistic understanding of freshwater systems and catchment status, health, and function.

## 1 Introduction

Recent technological advances in high-frequency water quality measurements have significantly shifted the state-of-the-art methods in several areas of catchment science, stream hydrochemistry, aquatic ecology, and freshwater and wastewater management. High-frequency water quality measurements facilitate the analysis of dissolved or suspended chemicals in water, spanning sampling intervals from seconds to hours and using a range of automated instruments deployed *in situ*: autosamplers, electrochemical probes, optical sensors, wet-chemistry analyzers, and lab-on-a-chip tools based on microfluidics and nanotechnology. The main advantages of these technologies is the matching of the sampling intervals of water quality measurements with the process rates of underlying hydrometeorological and biogeochemical drivers and the ability to obtain large amounts of water quality data in an automated and systematic way. Thus, high-frequency measurements can identify fine-scale temporal variation in water quality patterns and underlying processes that have been previously unrecognized or underappreciated using traditional low-frequency sampling approaches (weekly to monthly grab sampling for lab-based analyses). High-frequency data provide unprecedented information on coupling between hydrological, biogeochemical, and ecological processes controlling streamwater quality that can help improve routine water quality monitoring programs, e.g., by indicating appropriate sampling frequencies^1,2^ and locations,^3,4^ making monitoring easier, e.g., through proxies,^5,6^ cheaper,^7^ safer, or even possible during extreme events^8^ or in remote locations.^9^

**Figure 1:**
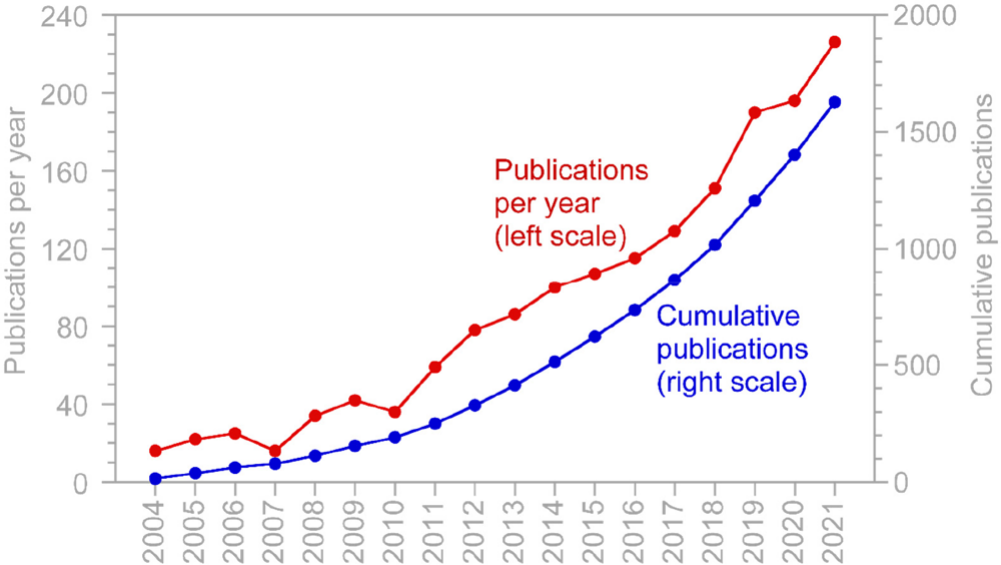
Number of peer-reviewed journal articles containing search phrases “high-frequency” or “high-resolution” and “water quality” in the title, abstract, or keywords. Based on a Web of Science search in August 2022.

Certain high-frequency water quality measurements have been available for many decades; e.g., Hydrolab’s multiparameter field water quality probe was use introduced in 1968 and Turner Model 10 field fluorometer in 1973.^10^ However, the unprecedented potential of high-frequency water quality measurements was first noted in the seminal paper of Kirchner et al.^11^ Since then, the “high-frequency wave”^10^ has continuously accelerated with a growing number of scientific papers being published each year (Figure 1) and four thematic international conferences held, in Magdeburg in Germany (2014), Sandbjerg in Denmark (2016), Clonakilty in Ireland (2018), and Uppsala in Sweden (2021). Recent developments in high-frequency water quality measurements have brought new insights into mechanistic understanding of abiotic and biotic catchment and stream processes which are increasingly used to evaluate the effectiveness of water monitoring and management efforts. Application areas so far include (1) evaluation of concentration–discharge relationships to identify solute/particulate mobilization and delivery patterns,^12−15^ (2) estimation of travel time distributions and identification of flow pathways in catchments using tracers,^16−19^ (3) development and validation of catchment hydrogeological and hydrochemical models,^20,21^ (4) improved estimation of pollutant loads and concentrations to comply with statutory requirements,^22,23^ (5) evaluation of diel cycles and estimation of stream metabolism based on dissolved oxygen (DO) sensors,^24−27^ (6) impact assessment of multiple stressors on stream biota^28,29^ and impact of stream biota on water quality,^30^ (7) evaluation of feedbacks between biogeochemical cycles and hydrology,^31,32^ (8) evaluation of trade-offs between different ecosystem services and management solutions,^4,33^ (9) development of proxies for difficult-to-measure water quality parameters based on readily available sensor data,^34,35^ (10) online water quality monitoring for drinking water and wastewater treatment optimization,^36,37^ and (11) combining high-frequency data with artificial intelligence tools to develop early detection systems for water contamination and algal bloom outbreaks.^38,39^

In this paper we explore and discuss the current state-of-the-art methods and potential future developments in high-frequency water quality measurements and their contributions to our understanding of hydrological, biogeochemical, and ecological processes. We focus on freshwater aquatic systems, mainly streams and rivers, in which flow discharge is the dominant underlying control of observed high-frequency water quality patterns. For a review of high-frequency applications in other types of aquatic systems, we refer the reader to specific reviews, e.g., on lake,^40^ marine,^41^ urban,^42^ and drinking waters.^43^ In contrast to previous reviews in this topic for streams and rivers, we focus on a wide range of water quality parameters rather than specific constituents, such as dissolved organic carbon (DOC)^44^ and chemical oxygen demand,^45^ nutrients^36^ including nitrate (NO_3_^–^),^46^ or specific technologies, e.g., UV–vis sensors.^47^ We build on a comprehensive feature article by Rode et al.^10^ and doubling of scientific articles published on this topic since 2016 to show how high-frequency water quality measurements further advance catchment science, hydrochemistry, and aquatic ecology by providing the unified understanding of catchment and streamwater quality patterns and processes across spatial (from individual stream reaches to diverse stream networks) and temporal (from storm events to seasons and decades) scales.

## 2 High-Frequency Water Quality Technologies

Existing and emerging high-frequency measurement technologies are outlined in Table S1 and include instruments and tools that allow high-frequency (subdaily), *in situ* and automated measurements of water quality constituents. These include both well-established technologies such as autosamplers, ion-selective and electrode-based sensors, and recently developed fully automated optical sensors based on UV–vis absorbance^48^ and fluorescence^49^ spectroscopy or wet-chemistry laboratories facilitating measurements of carbon (C), nutrients,^23,50^ major ions, and stable isotopes^18,51^ using miniaturized *in situ* or field-lab deployment of laboratory methods, e.g., ion chromatography.^52^ Existing high-frequency technologies enable measurements of a wide range of parameters, and all have specific advantages and limitations that are critical to consider before their deployment. High-frequency water quality instruments are designed for measuring specific parameters (e.g., NO_3_^–^, pH, DO, temperature) across a specific range and with a specific sensitivity. Thus, the choice of instrument (including its analytical limits of detection, precision and accuracy) might vary depending on the type of aquatic environment to be monitored (e.g., natural waters vs wastewater treatment plants, lotic vs lentic ecosystems) or specific water quality conditions (e.g., presence of high turbidity or color due to high concentrations of DOC). Some sensor technologies, such as ion-selective electrodes, were originally developed for measuring solutes in highly polluted waters, e.g., wastewater treatment plants. Nowadays, they are often replaced by optical sensors, which allow automated cleaning and improved detection limits and precision of measurements in natural waters.

Optical sensors, using absorbance- or fluorescence-based approaches, have revolutionized high-frequency water quality measurements over the past two decades. The absorbance-based sensors utilize ultraviolet and visible (UV–vis) spectrometry and can typically measure absorbance between 200 and 720 nm. Predefined spectral algorithms called global calibrations^48,53^ allow indirect estimation of NO_3_^–^ (at ∼200 nm), DOC (at ∼254 nm), and turbidity (at ∼700 nm). New global calibrations are continuously being developed and tested, e.g., for chlorophyll, but their accuracy typically depends on site-specific water chemistry and source-specific compound matrix. Therefore, manufacturers recommend establishing local calibration curves to account for local water quality conditions through parallel sensor deployment and grab sampling with analyses performed in laboratory. Fluorescence-based sensors, also called fluorometers, measure fluorescence intensity of dissolved organic matter (DOM) at a predefined combination of excitation and emission wavelengths. Fluorescence-based sensors typically include humic-like fluorescence (or peak C, excitation 365 nm, emission 480 nm) used as a DOC proxy^35,49,54,55^ and tryptophan-like fluorescence (peak T, excitation 280 nm and emission 340 nm) which represents the proteinaceous fraction of the DOM pool.^56^ Fluorescence-based measurements are both temperature and turbidity dependent, and thus, adequate correction algorithms are necessary.^35,53,57^

Nutrients, mainly phosphorus (P) and nitrogen (N), C, major ions, and stable isotopes can be measured with wet-chemistry analyzers,^8,23,50,51,58^ which often employ ion chromatography or reagent-based, colorimetric methods. These analyzers are usually placed in an insulated trailer or gauging station beside a stream, from which water is pumped and homogenized at specified time intervals before being forwarded to the analyzers for specific constituent determination. These are usually in-line systems that offer a single constituent determination at any given time, and different constituents are measured consecutively on either the same or successive water samples. The main advantage of the wet-chemistry analyzers is their ability to measure P fractions and major ions that are currently not possible to measure with optical sensors. Limitations include challenges with sample filtration (an independent filtering system must be implemented), a high level of maintenance needed to prevent and troubleshoot sampling and analysis problems (such as clogging or freezing of sampling lines), and high power requirements.^50,58^

Several emerging high-frequency technologies (Table S1), including lab-on-a-chip devices,^59^ nano sensors,^60^ DNA-based biosensors,^61^ and molecular biosensors,^62^ have applications in freshwater ecosystems that have yet to be explored, partly due to a lack of commercially available instruments for widespread application. These instruments have potential for future low-cost rapid *in situ* and real-time determination of a wide range of water quality constituents, e.g., heavy metals or harmful algal toxins including emerging contaminants,^62,63^ beyond what is available with optical sensors or wet-chemistry analyzers.

A growing number of studies identifies pressing challenges with instrument deployment and management of large quantity of high-frequency water quality data. Typical deployment challenges include the need for frequent instrument calibration, maintenance (e.g., cleaning the optical windows of sensors), and troubleshooting (e.g., removing blockage in sapling lines of wet-chemistry analyzers) leading to measurement errors.^8,10,23,50,64^ Data artifacts due to *in situ* sampling have been widely recognized and reported including quenching effects due to high fine sediment concentrations, temperature, or biofouling (“sensor drift”) and require appropriate quality control/assurance protocols, typically developed by individual research teams. The quality of high-frequency water quality data sets critically impacts their ability to bring new scientific insights. Thus, we urge the scientific community to develop robust deployment, maintenance, and data management protocols.

### 2.1 High-Frequency Data Availability

We compiled a list of high-frequency water quality data sets and repositories that are either open access or available on request (Table S2). Most of the data sets are collected at high frequency for individual streams and catchments; by contrast, high-frequency data sets at high spatial resolution (e.g., multiple or nested catchments) are rare. Some of these data sets span more than a decade of measurements (see, e.g., ref (65)) but are mostly constrained to the northern hemisphere. Most of the open access data sets are generated by large research projects and sampling initiatives (e.g., the National Ecological Observatory Network NEON)^66^ that have both staff and resources to promptly quality ensure/control and publish high-frequency hydrochemical data. High-frequency data sets generated by smaller projects often take longer to publish open access but are available upon request. “Simpler” hydrochemical data sets (e.g., specific conductivity or temperature) are generally available as open access compared to “complex” data sets (e.g., nutrients, sediments, or stable isotopes requiring longer and more detailed validation). Furthermore, optical sensor nutrient and sediment data (e.g., NO_3_^–^ or turbidity) are more commonly available as open access compared to P fractions derived from wet analyzers. Several data clouds and repositories are currently available for storage of high-frequency hydrochemical data (e.g., HydroShare); however, there are challenges associated with aggregating data sets with inconsistent protocols used for data collection, maintenance, quality control, and accuracy in relation to the FAIR data principles.^67^ Since many high-frequency water quality data sets are not part of routine monitoring (e.g., for compliance with the Water Framework Directive or Clean Water Act) but are managed by individual research groups, we encourage efforts to design joint data quality assurance and control protocols for these data sets.

## 3 Exploring the Full Potential of High-Frequency Water Quality Measurements

**Figure 2:**
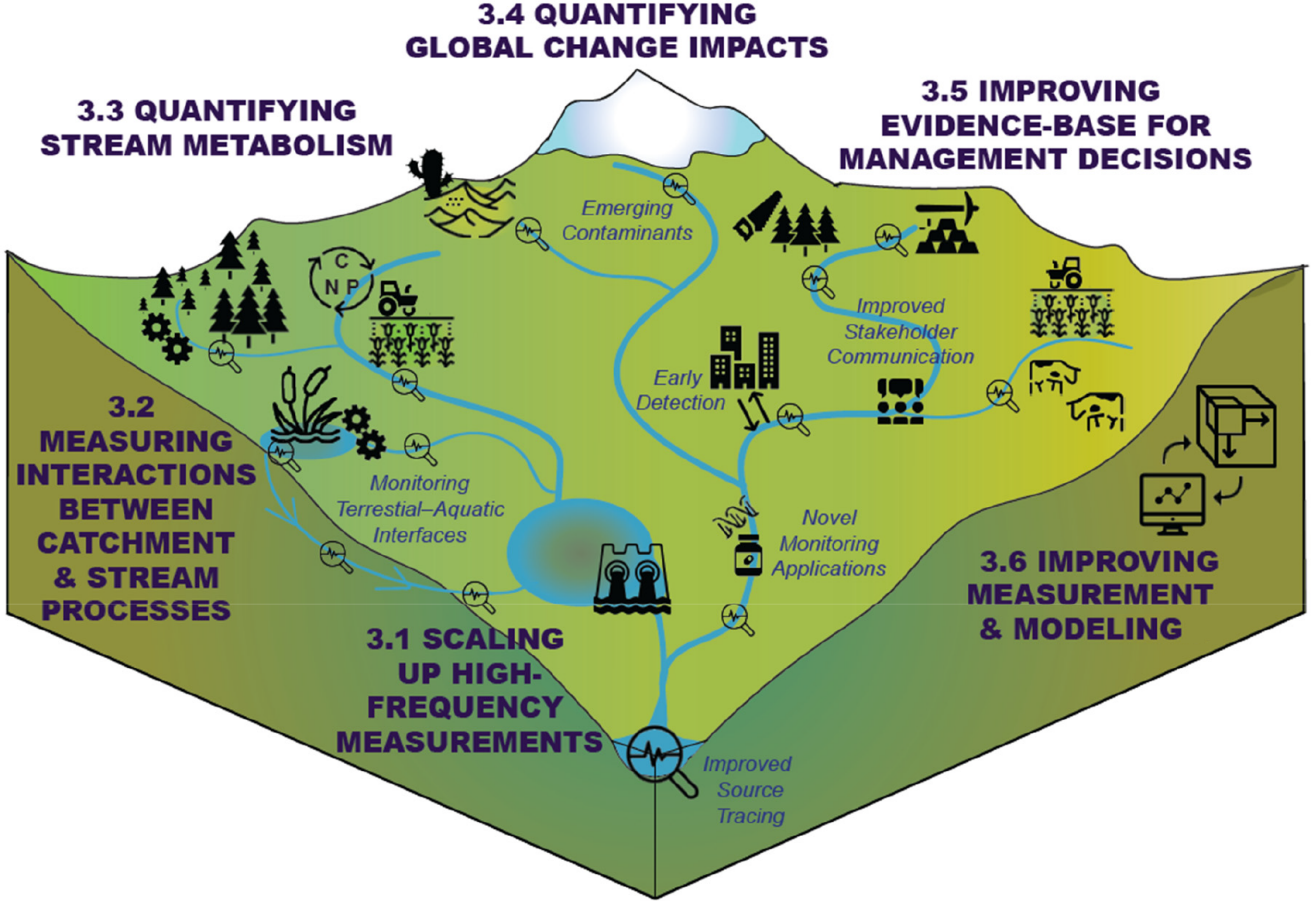
Six focus areas reviewed in the paper where high-frequency water quality monitoring contributes to significant scientific advancements within catchment science, stream hydrochemistry, and aquatic ecology and can lead to major improvements in freshwater quality. All icons were reproduced from https://icons8.com/icons/.

We have identified six areas where high-frequency water quality monitoring can contribute to significant scientific advancements and lead to major improvements in freshwater management (Figure 2). For each focus area, we provide both an overview of the scientific findings to date and specific examples of future scientific needs to enable further insights into the functioning of freshwaters and their catchments together with practical solutions for their protection.

### 3.1 Scaling Up High-Frequency Water Quality Measurements

**Figure 3:**
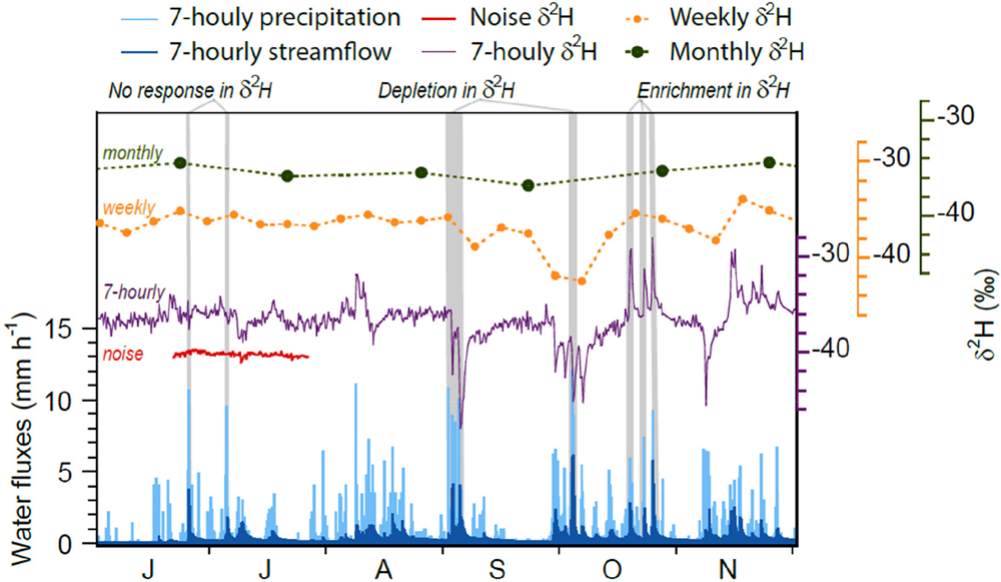
Six months of streamwater deuterium isotope ratios sampled at monthly, weekly, and 7-h frequencies (green, orange, and purple, respectively), compared to 7-h precipitation and streamflow water fluxes (light and dark blue, respectively) at Upper Hafren, Plynlimon, Wales (data of ref (19). Weekly sampling was simulated by resampling the high-frequency record every Wednesday at noon; monthly sampling was simulated by resampling at noon on the fourth Wednesday of each month.). Replicate analyses of a quality control standard (red) illustrate the noise level in the high-frequency measurements, showing that the fluctuations in the high-frequency isotope time series are mostly signal rather than noise. Coupling between hydrology and water quality dynamics is clearly visible in the high-frequency isotope record but is obscured by conventional weekly or monthly sampling.

High-frequency water quality measurements allow us to identify fine-scale fluctuations in stream chemistry in response to streamflow and “hot moments”^68^ of solute and particulate export (Figure 3). However, most of these measurements are recorded for single locations within a stream network, which complicates the generalization of reach-specific hydrochemical and biogeochemical patterns to entire catchments. High-frequency water quality patterns can be confounded by the influences of upstream contributing catchments and the overall catchment heterogeneity, representing a source of uncertainty when inferring solute/particulate sources and transport pathways and determining their underlying control mechanisms.^69^ Catchment heterogeneity is driven by a combination of static (geology, soil type, topography, geomorphology, land use) and dynamic hydroclimatic factors (precipitation and flow patterns) that govern the spatial and temporal distributions of hydrological responses, from storm events to seasons (see, e.g., ref (70)). These catchment heterogeneities contribute to complex responses in concentration–discharge (*c*-*q*) relationships and source–sink and source–distance relationships, which affect transport time and mode as well as in-stream biogeochemical transformations.^14,31,71,72^ We propose that spatially diverse catchment processes can only be unraveled if water quality sampling is carried out at both high temporal and spatial resolutions. This can be attained through upscaling high-frequency monitoring to cover multiple catchments (Section 3.1.1), combining high-frequency monitoring with low-frequency nested monitoring at the catchment scale to identify different source and transport pathways for solutes/particulates (Section 3.1.2), or monitoring water quality at high frequency at key ecohydrological interfaces to determine their impact on biogeochemical processing of solutes and particulates (Section 3.1.3).

#### 3.1.1 Patterns across Multiple Catchment Scales

Recent initiatives have led to establishing high-frequency water quality monitoring across multiple catchments,^65,73−75^ providing new opportunities to compare hydrochemical patterns and processes between catchments. For example, in the northeastern U.S., high-frequency monitoring of multiple catchments revealed similarities in nutrient export dynamics at the event scale for urban and forested catchments but variable responses for agricultural catchments explained by different land management practices.^76−78^ Further, NO_3_^–^ and DOC event export dynamics in catchments with diverse characteristics showed asynchronous behavior, suggesting that spatially distributed sources and antecedent conditions decouple solutes’ responses at both event and seasonal scales.^73^ Using data from 26 high-frequency monitoring sites in the Mississippi River Basin, U.S., Marinos et al.^12^ showed that arable fields with higher tile drainage densities consistently contributed to chemostatic responses of NO_3_^–^ (concentrations are strongly buffered and do not change with flow). Beyond the findings that catchments dominated by agricultural land use display higher variability in solute and particulate transport dynamics, compared to urban and forested catchments, Seybold et al.^78^ suggested that intercatchment monitoring could be further used to disentangle the effect of land use from other water quality controls, e.g., geology, geomorphology, and climate.

The increasing number of high-frequency water quality monitoring sites worldwide offers new opportunities to perform multicatchment comparisons at national, continental,^66^ or even cross-continental scales. Such efforts could greatly improve our understanding of large-scale water quality controls under short-term hydroclimatic fluctuations and long-term environmental change. Globally distributed high-frequency monitoring networks could also help to explain and synthesize the great variation in observed hydrochemical patterns and governing processes^66,79^ that in turn can lead to development of typologies of catchment and solute/particulate behavior^80^ and constrain contributions of riverine processing at continental scales.^81^ Remote sensing tools offer another opportunity for improving spatial resolution of high-frequency measurements. Current remote sensing applications include the determination of optical water quality properties of chlorophyll *a*,^82^ suspended solids,^83,84^ and DOC;^85^ these can be also measured at high frequency. Utilizing high-frequency water quality data sets in remote sensing applications can improve calibration of remote sensing data from small water bodies and headwaters^86^ and help to up scale high-frequency measurements from individual catchments to whole river basins.^84^

#### 3.1.2 Patterns along Stream Networks

Nested catchment and synoptic longitudinal sampling are established approaches to investigate longitudinal changes in water quality linked to different stream or subcatchment characteristics.^4,13,87,88^ When combined with high-frequency water quality measurements, nested catchment or synoptic sampling can provide new insights into catchment controls of solute and particulate behavior. For example, stream networks can be monitored by situating high-frequency instruments at points that receive water inputs from different landscape compartments.^4,89^ This approach allows detection of differences in how subcatchments or even individual fields in arable catchments contribute to streamwater chemistry at a given point in time (synoptic sampling) or over time (if high-frequency water quality instruments are deployed over an extended period). The nested catchment approach can help scale the inferences drawn from point measurements to large, complex catchments by untangling confounding factors.^13,75^ Using synoptic high-frequency discharge and NO_3_^–^ concentration data, Winter et al.^13^ showed that different subcatchments can have seasonally variable contributions to storm event dynamics; this observation would have been overlooked with low-frequency data or even high-frequency data from a single monitoring station. Combined, low-frequency nested monitoring and high-frequency sampling at selected stations can improve the identification of hot spots and hot moments of solute and particulate generation.^68,87,90^ These methods can be used to differentiate transport–time versus transport–distance relationships, fine-tune catchment-scale solute and particulate flux and yield estimates,^91^ and track contaminant plumes to assess the spatial extents at which specific disturbance events (e.g., storms, fires, mining incidents, construction) have influenced water quality.^91,92^

Moreover, high-frequency data loggers or sondes can be mounted on boats, buoys, autonomous underwater vehicles (AUVs), or unmanned aerial vehicles (UAVs, e.g., drones) to measure hydrochemistry synoptically across transects or cross sections^93^ or along a river or stream reaches,^3,87,94^ allowing for Lagrangian sampling (following a parcel of fluid as it moves) in contrast to traditional fixed sampling sites (Eulerian sampling). For example, Chen and Crossman^95^ installed wet-chemistry analyzers onto floating devices to capture P dynamics in large rivers, and Hensley et al.^3^ used a suite of sensors attached to a river raft for Lagrangian water quality profiling. Lagrangian sampling utilizing high-frequency water quality measurements has significant potential for new hydrochemical discoveries as it enables capturing the evolution of water quality signals in stream networks to identify key source and transport pathways for solutes^96^ and particulates.^4^ A recent cutting-edge development is using AUV swarms (multiple small AUVs equipped with sensors) to communicate, locate, and home-in on target areas or water quality issues, e.g., toxic algal blooms.^97^

#### 3.1.3 Patterns at Key Ecohydrological Interfaces

At a finer spatial scale than subcatchments, certain key hydrological interfaces can function as environmental control points for solute and particulate export.^98^ The convergence of source and hydrological transport controls means that these zones exert disproportionate control over solute and particulate export. Environmental control points are often found at the intersection of hydrological flow paths at land–water interfaces, such as hyporheic and riparian zones.^68,99^ They are central in controlling water quality in the stream network due to the mixing of different waters (groundwater/overland runoff and surface water) with different chemical compositions that can enhance rates of biogeochemical reactions and microbial activity, e.g., due to accumulation of labile C sources.^100^ Investigating solute processing mechanisms in such key areas can be advanced by high-frequency monitoring that captures the temporal dynamics of hydrological and biogeochemical processes simultaneously. This approach was used by Werner et al.,^101^ who measured in-stream DOC concentrations at high frequency combined with a small-scale topographic analysis of the riparian zone in a forested headwater catchment in Germany. They showed that a small pool of DOC from local depressions that only represented 15% of DOC in the riparian zone contributed to 85% of the total DOC export to the river network. However, most studies that integrate high spatial resolution monitoring at key interfaces have not done so at high temporal resolution. As such, only a snapshot of certain conditions is detectable, and critical hot moments of solute export (e.g., first-flush storm events during fall^15^) are likely to be overlooked. Increasing our efforts to understand the environmental control points for water quality in both space and time (facilitated by denser or more targeted networks of high-frequency water quality measurements) could be a large step forward to reveal water quality controls, contributing to protection and restoration of water quality at the catchment scale.^80^

### 3.2 Interactions between Catchment and Stream Processes

Despite our growing knowledge of nutrient transformations and particulate transport linkages in aquatic systems,^102,103^ the use of high-frequency water quality measurements to assess the role of terrestrial–aquatic linkages in long-term nutrient and organic matter cycling is an emerging field of research. Under the River Network Saturation concept, a system’s ability to regulate the processing of increased organic material or solute concentrations under high-flow conditions is dependent on the rates of *in situ* processing and inputs from the hydrologically connected terrestrial and aquatic environments.^32^ Use of high-frequency sensors provides an opportunity to track how solute and particulate fluxes change along the land–water continuum in natural vs impacted freshwater systems, facilitating tests of classical hydrochemical concepts (river continuum,^104^ nutrient spiraling,^105^ chemostat,^103,106,107^ pulse-shunt,^108^ river network saturation,^32^ and allometric scaling of riverine biogeochemical function^81^), and enabling comparisons of *in situ* solute transport and cycling dynamics to solute addition experiments in systems ranging from small headwaters to big rivers.^32,109^ Unlike early solute addition experiments targeting only baseflow conditions, high-frequency water quality measurements enable estimation of nutrient and organic matter transport and cycling at a wide range of flows.^31,110^ The inclusion of high-frequency monitoring data in such estimates can strengthen our understanding of the role of streams and rivers in transporting and processing solute and particulate fluxes,^81^ integrating different biogeochemical cycles over a range of time scales and environmental conditions.

#### 3.2.1 Concentration–Discharge Relationships

Combined time series of high-frequency water quality and discharge have elucidated hydrological and biogeochemical controls of water quality using *c*-*q* relationships.^111^ The slope between concentration and discharge on the log–log scale (the *c*-*q* slope) is a common metric to characterize solute or particulate export.^106,112,113^ The hydrochemical behavior of running waters in this context can be conceptually understood as chemostatic (little change in concentration as discharge varies) or chemodynamic (hydrological flushing resulting in concentration or dilution behavior). On the event scale, *c*-*q* slopes enable fingerprinting of catchment sources and pathways for solutes and sediments which cannot be elucidated from *c*-*q* patterns determined with low-frequency sampling.^13,14,71^ Hysteretic *c*-*q* patterns, resulting from different rates of change for concentration and discharge between the rising and falling limbs, provide information on the timing of concentration response in relation to the hydrograph and can be further analyzed to infer solute and particulate mobilization and delivery patterns.^71,114^

Despite efforts to systematize catchment-specific *c*-*q* patterns, robust classification is notoriously difficult as it depends on variable storm event characteristics and antecedent conditions that often produce unique concentration responses.^114^ A common way to summarize storm event *c*-*q* patterns is to calculate hysteresis and flushing indices that quantify the magnitude and direction of the *c*-*q* hysteresis^115^ and concentration/dilution behavior^77,114^ respectively. Often these metrics are used in combination (see, e.g., refs (31, 77, 116)) to detect *c*-*q* patterns across temporal scales, e.g., from individual storm events and seasons to hydrological years and decades^14,117^ and across different catchments.^118^ Most studies of *c*-*q* patterns from high-frequency measurements have primarily focused on event-driven hydrological flushing patterns (concentration or dilution) with fewer studies investigating *c*-*q* relationships for low and stable flow conditions. There is a growing need to provide robust conceptual models of stream *c*-*q* relationships across spatial and temporal scales, necessitating high-frequency water quality measurements in a range of catchments over long time spans.

#### 3.2.2 Interactions between Biogeochemical Constituents

Biogeochemical cycles in aquatic environments rarely occur in isolation, leading to potentially complex interactions between biogeochemical constituents in both space and time. For example, monitoring the temporal variability in C, N, and P concentrations and C:N:P stoichiometry in freshwaters can provide critical information about nutrient limitations in primary production, nutrient-driven water quality impairments, and eutrophication risks.^25,27^ Microbial demand for C, N, and P follows their approximate molar ratios in biomass,^119^ but in-cell metabolism causes a delay in P uptake, which might be better resolved with high-frequency measurements. Global change, especially increasing agricultural land use, has altered these ratios and therefore influenced the turnover of C, N and P in many catchments.^120^ Further, hydrologic processes changing with climate can cause shifts in the stoichiometric C:N:P ratios and thereby shifts in limiting nutrients.^121^ As these effects can be operating both on small scales and over short time spans, high-frequency measurements could yield in-depth knowledge of combined nutrient and C turnover.

High-frequency measurements provide information about concentrations of nutrients (total P [TP], reactive P, total N, NO_3_-N) and C (DOC, total organic carbon [TOC]) but also in-depth information on DOM quality and its biogeochemical interactions, e.g., the complexation of DOM with metals such as mercury.^122^ For example, Wilson et al.^55^ used humic-like fluorescence (peak C intensity) as an indicator of biodegradable DOC and dissolved organic nitrogen concentrations. Bulk DOC concentrations alone cannot describe highly variable organic matter composition, but absorbance measurements at specific wavelengths and their ratios can provide additional information on DOM quality. For example, specific UV absorbance at 254 nm (SUVA_254_) is used as an indicator of DOM aromaticity, and spectral slope ratio (S_R_) and E4/E6 ratio are often used as indicators of DOM molecular weight.^123^ Similarly, fluorescence-based measurements can provide information on DOM origin (fluorescence index) and age or transformation (humification index and biological index).^57,124^ Peak T or tryptophan-like fluorescence has been used to estimate a range of water quality parameters such as biological oxygen demand,^35,49^ chemical oxygen demand,^125^ and *E. coli*.^126^ The coupling of high-frequency nutrient and DOM measurements can further elucidate processes regulating DOM dynamics and their interactions with other biogeochemical cycles in aquatic ecosystems.

### 3.3 Quantifying Stream Metabolism in Freshwater Systems

The study of biogeochemical processes in streams has long been constrained by difficulties in monitoring spatially heterogeneous and temporally dynamic processes in larger, nonwadeable streams and rivers during challenging weather conditions. Consequently, estimates of metabolic rates in these aquatic systems are often biased toward sunny, low-flow conditions, failing to capture the impact of hydrologic disturbances and nutrient pulses.^127^ With the advent of high-frequency monitoring, new possibilities have emerged to address the knowledge gaps of predictive metabolic patterns and nutrient cycling in streams across wider spatial and temporal scales.^98,128^ The development of multiparameter sensors has helped to catalyze studies that merge stream ecology with hydrochemistry and catchment science and allowed for a more fine-grained partitioning of biogeochemical processes during critical times for solute and sediment export. The improved understanding of in-stream processing regimes ensures better predictions for both nutrient loading and greenhouse gas fluxes, which can be used to inform management decisions about expected impacts of land use, flow regulation, and stream restoration.

#### 3.3.1 Measuring Nutrient Cycling and Disturbance Effects

Initial sensor-enabled studies have often focused on hydrochemical dynamics and the relationships between solutes and discharge in aquatic ecosystems.^10^ Advances in ecosystem production modeling,^129^ and the emergence of long-term high-frequency data sets including nutrient, oxygen, and hydrologic data, have provided new insights into streams’ metabolic capacities to process nutrients under differing hydrological conditions, over time series that now span multiple years or even decades. Biological processes such as autotrophic assimilation, ecosystem respiration, and denitrification respond to changes in diel light and temperature patterns.^110,130^ These processes are also highly dependent on hydrology and can be linked to nutrient uptake and release. Previous constraints with discrete and labor-intensive measurements of metabolic activity have now been surpassed by high-frequency water quality measurements that provide data needed for modeling of metabolic processes throughout seasons and for all flow conditions.^131^ For example, Jarvie et al.^33^ used high-frequency monitoring to understand the metabolic controls of P and N release in a pristine wetland. During periods of low flow, an increase in primary production provided abundant C sources that in turn fueled microbial respiration and mineralization of bioavailable nutrients. This sequence ultimately led to sustained high-intensity nutrient release events where ammonification of NO_3_^–^ further enhanced phosphorus release.^33^ The impact of hydrology on nutrient cycling has been shown to depend on the magnitude of storm events in two studies using modeling^108^ and data mining approaches.^31^ High-magnitude storms can suppress diel nutrient cycling by reorganizing benthic substrates,^108^ while diel cycling behavior can persist during low-to-moderate magnitude storm events and rapidly re-establishes (∼12 h) after high-magnitude storm events.^31^ Although these findings require further validation using *in situ* biogeochemical and tracer data, they can help to conceptualize nutrient behavior and the interlinked nature of hydrological and biogeochemical processes in streams and rivers.

High-frequency measurements of DO and NO_3_^–^ can be used to partition stream metabolic pathways responsible for N transformations by coupling NO_3_^–^ concentrations with the net autotrophy–heterotrophy ratio. This advance has generated new insights into the importance of light availability, hydrology, and nutrient limitation to in-stream processing of N^25,27,132^ and P.^133^ Continuous sensor deployments can reveal seasonal shifts in in-stream processes as well as impacts of hydrological disturbances. For example, in a groundwater-fed river in Arkansas, U.S., there was a shift from autotrophic assimilation to denitrification and increasing NO_3_^–^ removal efficiency when discharge returned to baseflow after a period of storm events.^25^ During hydrologically active periods in spring and early summer, with high primary production, oxygenated water entered the hyporheic zone and favored aerobic nitrification while suppressing denitrification, resulting in a net production of NO_3_^–^ along the river. During the late summer and fall period with lower flows, there was a decline in primary production, an increase in microbial decomposition of organic matter, and depletion of available oxygen favored denitrification, thereby converting the stream to a net NO_3_^–^ sink. In this system, water residence time and seasonal shifts in stream metabolism were the primary factors linked to NO_3_^–^ removal and would have been undetected if not for high-frequency water quality measurements.

#### 3.3.2 Linking Stream Metabolism with Carbon Dioxide Emissions

The ecological implications of stream networks as biogeochemical hotspots are, apart from nutrient cycling, further manifested in the global C cycle. Stream CO_2_ efflux is controlled by biological processes that follow diel patterns,^69,130^ but substantial proportions are often originating from terrestrial respiration with subsequent subsurface transport to streams. By combining high-frequency data sets of stream metabolic parameters and subsurface flow pathways, an improved understanding of the sources, dynamics, and magnitude of C efflux from aquatic systems can be achieved. Fluxes of C from running waters are increasingly recognized as important components in the global C cycle, as rivers and streams are often supersaturated with CO_2_, with partial pressures largely exceeding that of the atmosphere.^134,135^ Estimates of global CO_2_ emissions from streams and rivers to the atmosphere are at present 3.48 PgC yr^–1^ but have been systematically adjusted upward as monitoring technology has improved and expanded across biomes.^136^ Despite improvements in accuracy, a major source of underestimation in emissions can be attributed to manual sampling that is often biased toward daytime, failing to capture the diel nature of CO_2_ efflux. Photosynthetic assimilation of CO_2_ peaks at noon, which lowers the CO_2_ concentrations in the water column, whereas during nighttime, stream concentrations of CO_2_ increase due to the dominance of heterotrophic respiration. Based on a global data set of high-frequency pCO_2_ measurements, the potential deficit of nighttime CO_2_ emissions was modeled on discrete daytime samples that constitute the basis for current global estimates.^137^ The model revealed an unaccounted additional contribution of 27% from nocturnal CO_2_ emissions. A similar study conducted in Europe estimated the nocturnal deficit at 39%.^138^ This demonstrates that rivers and streams may be important sources of CO_2_ and other greenhouse gases, e.g., methane (CH_4_) and nitrous oxide (N_2_O); however, high-frequency measurements of their concentrations are to date limited.^139^. Moreover, these studies highlight a blind spot that arises when only discrete daytime measurements of CO_2_ are made and show that diel cycles in autotrophic and heterotrophic processes in streams need further consideration, necessitating continuous high-frequency *in situ* monitoring.

### 3.4 Quantifying Global Change Impacts on Freshwater Systems

High-frequency water quality monitoring can be a useful tool to detect impacts of global change on aquatic systems and to understand emerging changes in water quality and stream biogeochemistry due to changing pressures and stressors. Current high-frequency water quality monitoring in the perspective of global change often focuses on assessing the influence of direct (e.g., agricultural practices, urban development) and indirect (e.g., changing precipitation patterns, thawing permafrost, melting glaciers, desertification, wildfire) anthropogenic impacts.^140,141^ Key topics include detection of changes in hydrologic and biogeochemical drivers, identifying changing patterns in baseflow and stormflow solute and sediment transport, and documenting shifts in ecosystem function (e.g., stream metabolism). Studies in agricultural and urban catchments often focus on the direct results of land-use change on water quality and quantity such as capturing short-duration events (e.g., drought,^142^ flash floods) or understanding the mobilization of road salts into streams during and after storm events in urban settings.^143^ Though the effects of direct global change are well documented for human-impacted systems in the temperate and boreal climatic zones, less attention has been focused on regions experiencing accelerated environmental change, such as the polar and cold regions,^9,144^ as well as regions where long-term monitoring is not currently feasible (e.g., regions experiencing civil unrest). High-frequency water quality measurements could help to improve spatial coverage of water quality data sets in these regions and advance the mechanistic understanding of global long-term change patterns.^98,116,145^

As urban and agricultural areas undergo extensive land-use change, forest fire rates increase, and permafrost and glacial landmasses thaw, legacy and emerging contaminants will be released to downstream aquatic systems.^91,140,146^ Likewise, legacy nutrients released with land-use change are also a rising water quality concern worldwide,^33,147^ and when combined with higher storminess of changing climate, they can mask water quality improvements due to management efforts.^148,149^ Readily available long-term high-frequency data (Table S2), can be used to look at nutrient cycling in relation to past loading (e.g., the effect of nutrient legacies in agricultural landscapes) and to predict its future trajectories and feedbacks, particularly in response to environmental change. Further research efforts should place an emphasis on developing concentration–proxy relationships with established sensor technologies for emerging chemicals of concern (e.g., nitrapyrin, polychlorinated biphenyls, polycyclic aromatic hydrocarbons, perfluorochemicals) in both human-impacted and reference catchments. Autosamplers can be used to monitor changes in contaminants for which there are currently no high-frequency optical sensors or established proxies available (e.g., microplastics, pesticides, *E. coli*, salmonella).^140,150^ Future development of proxies or sensors (e.g., biosensors, nanosensors) for trace metals and other contaminants is important for informing best management practices aimed at the restoration or remediation of impacted freshwater systems.^151^

### 3.5 Improving the Evidence Base for Water Quality Management

**Figure 4:**
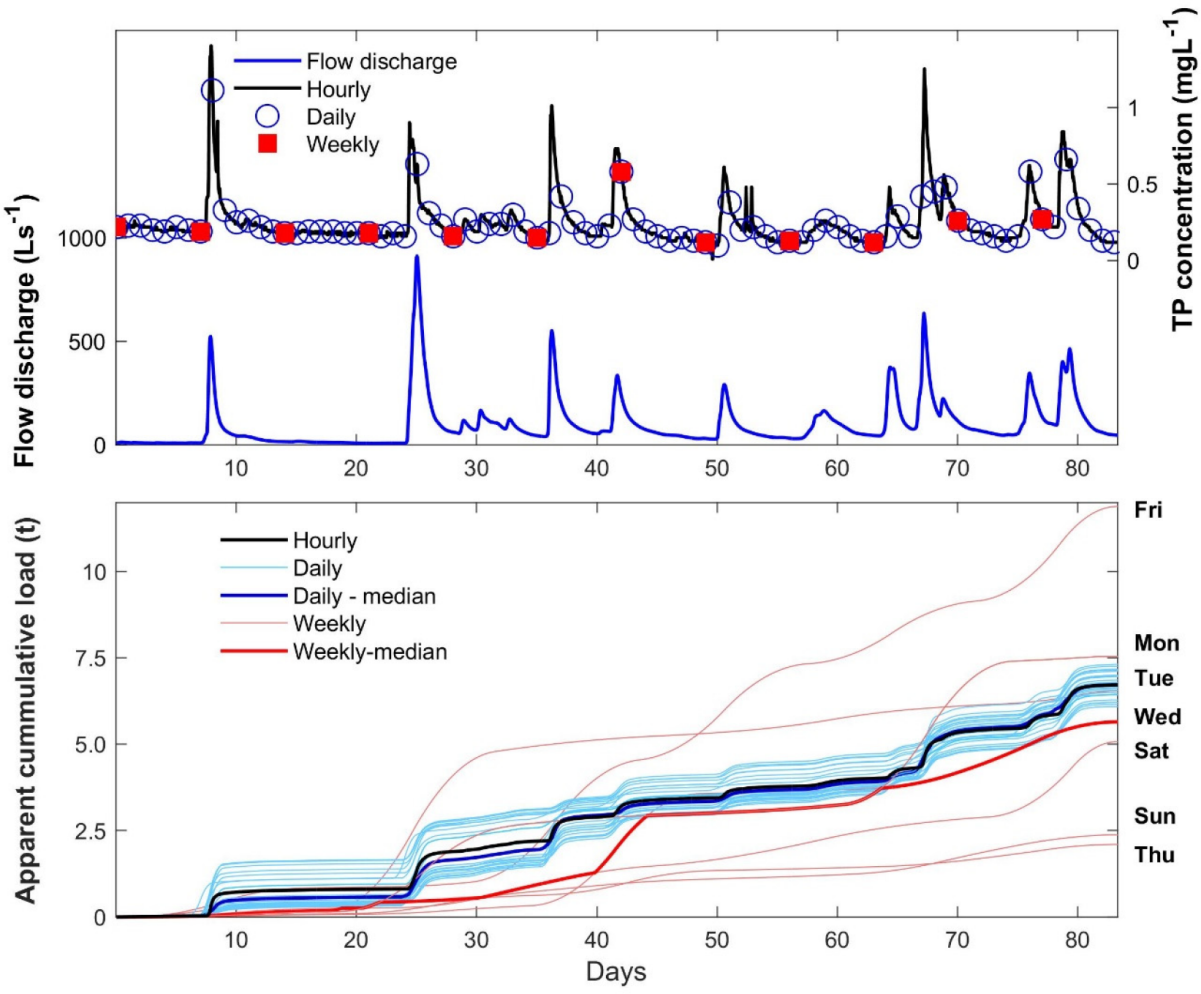
Effect of sampling frequency on load estimation. Top graph shows time series of flow discharge (blue solid line) and total phosphorus (TP) concentrations at hourly (black solid line), daily (blue circles), and weekly (red squares) sampling intervals. Hourly TP concentrations follow directly variation in flow discharge indicating a close coupling between flow generation and TP mobilization and delivery. Depending on sampling routine (time of day and day of week), daily and weekly TP concentrations often do not capture the actual range of concentrations measured with hourly sampling. This is particularly visible during storm events. Bottom graph shows variation in apparent cumulative TP load depending on the sampling frequency. Actual load based on hourly measurements is shown as black solid line. We simulated 24 daily sampling routines (light blue) corresponding to sample collection every day at the same time, e.g., every day at 12 pm. We also simulated seven weekly sampling routines (light red) corresponding to sample collection every week on the same weekday at noon, e.g., every Monday at 12 pm. Median values for daily (dark blue, partly obscured by black line depicting actual loads) and weekly (dark red line) loads were also calculated. Relative errors compared to actual loads varied for daily sampling from −10% (12 pm) to 9% (4am) with a median value of −0.02% and for weekly sampling from −69% (Thursday) to 77% (Friday) with a median value of −16%. High-frequency data were derived from Bieroza et al.^4^

High-frequency water quality monitoring, open-source big data, and machine learning approaches are converging to inform environmental policies related to water quality management and ecohydrology. For example, Kirchner and Neal^152^ used both high-frequency and long-term monitoring data from Plynlimon, Wales, to demonstrate that solutes spanning the periodic table all have non-self-averaging time series. Such time series confound naïve statistical expectations that averages should become more stable as one collects more data; instead, monthly, yearly, or decadal averages are approximately as variable, one from the next, as individual measurements taken hours or days apart. This further implies that catchment storage, transport, and mixing can generate visually and statistically convincing trends in surface water quality, on all time scales, which may be impossible to distinguish from trends arising from biogeochemical processes and which may be highly unreliable as predictors of future trends. This behavior presents obvious challenges for water quality management and deserves further study. In regulatory monitoring programs, there is typically a trade-off between spatial coverage and frequency of monitoring. In these types of applications, one of the major needs now are low-cost sensors that could be deployed as sensor networks across a wider spatial coverage to increase temporal resolution of water quality data, help to discover persistent patterns such as non-self-averaging behavior and pollution risks, and help guide water management decisions over multiple time scales, e.g., by allowing stakeholders to respond dynamically to changes in water quality in order to ensure quality of drinking water, protection of aquatic life, and human health. High-frequency water quality measurements can provide information on adequate sampling frequencies needed to accurately predict mean concentrations or loads (Figure 4) for different pollutants^1,7,50^ and catchment types, e.g., using concentration–discharge relationships.^113^ Thus, low-cost sensors that can be deployed directly in streams with minimal power, and reagent requirements could improve current environmental monitoring programs for detecting baseline conditions or testing specific hypotheses, e.g., for the purpose of operational monitoring.

High-frequency monitoring data can enable river managers to implement tailored and cost-effective solutions to fulfill the requirements of regulatory monitoring frameworks such as the European Water Framework Directive (WFD), the European Nitrates Directive, the Australian Water Act, and the U.S. Clean Water Act (CWA).^153^ For example, high-frequency monitoring is used in six Irish agricultural catchments within the Agricultural Catchments Programme (ACP) to evaluate impacts of agricultural land use on water quality and ecology.^23^ The ACP efforts show that high-frequency water quality data facilitate an improved understanding of how the catchments respond to changing weather conditions and agricultural practices, including impacts of climate change and mitigation measures.^29,65,154^ It serves also as a successful example for informing upcoming monitoring programs in other countries, e.g., in Denmark^155^ and Finland.^156^ Within Australian water quality monitoring, mostly discrete point-based sampling is conducted with selected river gauging stations measuring basic water quality parameters such as temperature, electrical conductivity, pH, and DO at high frequency.^157^ These efforts show that combining high-frequency measurements with statistical models could revolutionize monitoring programs and increase scientific understanding of spatiotemporal dynamics associated with climate change and hydrological variabilities.^157,158^ In the U.S., to understand long-term changes in water quality as a result of the CWA, the National Ecological Observatory Network (NEON; Table S2) was designed specifically to provide open-access high-frequency data from research sites spanning different climates and habitats with 34 stations out of 81 specifically targeting freshwater systems.^66^

Despite the efforts made, the implementations of WFD in EU member states, Water Act in Australia, and CWA in the U.S. have not yielded significant improvements in the ecological status of aquatic systems. It has been argued that the lack of success depends on limitations in scientific understanding (e.g., fragmented understanding of feedbacks between pollution sources and impacts), methodology,^159^ and implementation (e.g., insufficient stakeholder involvement^160^). High-frequency water quality data can fill the scientific gaps related to, e.g., understanding variation in catchment response to human impacts,^161^ determining water quality thresholds,^8^ or quantifying linkages between chemical and ecological status and pollution sources and ecological impacts. Further, it can be used to engage stakeholders in catchment programs, e.g., through visualizing how land management activities, such as fertilizer applications, relate to nutrient transport in streams,^162^ or through citizen science projects on water quality.^96^ van Geer et al.^153^ stressed the importance of high-frequency monitoring as part of the routine workflow of environmental agencies engaged in groundwater and surface water quality management. They predicted that long time series will become necessary to assess trends over longer periods of time, requiring robust systems for data storage, quality assurance, and control, as well as open-access data availability. Here, we envision integration of high-frequency water quality measurements into existing monitoring programs, together with more active stakeholder engagement as an important step toward improving water quality under changing environmental conditions and more stringent requirements, e.g., from the UN Sustainable Development Goals or the EU Green Deal.^80^

### 3.6 Combining High-Frequency Water Quality Measurements with Statistical and Modeling Tools

Kirchner et al.^11^ predicted that high-frequency chemical data will open the door to new applications of statistical and data-driven approaches for data reduction and pattern detection such as spectral analysis, wavelet techniques, and cross-spectral and cross-correlation analyses. Our ability to fully utilize the potential of high-frequency data depends on efficient analytical approaches to detect and distinguish noise (e.g., due to sensor malfunctioning) from underlying hydrochemical patterns and identifying drivers and complex interactions with environmental variables.

High-frequency measurements are often analyzed using time series statistics to infer evolution of chemical signals over time, detect periodic events, and identify hot moments of solute/particulate transport. One such approach is spectral decomposition for frequency-dependent water quality fluctuations, i.e., decomposition of variations in concentrations into frequencies and their related amplitudes.^152,163^ Frequencies with high amplitudes are often linked to periodic processes such as seasonal or diurnal cycles^164^ that can be linked to biogeochemical drivers of solute and particulate stream signals.^31^ To visualize shifts in frequency-dependent fluctuations over time, wavelet transforms are often used to analyze high-frequency water quality data.^38,165,166^ The wavelet coherence metric is a way to relate wavelet transformations of different variables during specific months or seasons and can therefore capture interactions (e.g., a relation between a chemical variable and discharge).^166^ Wavelet methods can also play an important role in analyzing time series that are unevenly sampled, whether by design, through equipment failures, or due to extreme conditions (e.g., during seasonal drought or water column freezing). Such sampling gaps are an inherent feature of high-frequency data sets^8,23,50^ and can lead to analysis artifacts due to leakage of spectral power from strong low-frequency signals. Specialized wavelet methods^152,167^ can help to reduce this leakage and thus suppress the associated spectral artifacts.

Because of the complex interactions between hydrochemical parameters measured at high-frequency, machine learning approaches are often used to identify patterns in high-frequency data or to detect any data interdependencies, through unsupervised or supervised learning.^168^ Machine learning approaches have been used to automate identification and correction of anomalies in high-frequency water quality data^169^ and estimate nutrient concentrations from high-frequency UV–vis absorbance,^170^ fluorescence,^171^ DO, specific conductivity, and turbidity measurements.^172,173^ Fewer studies have used machine learning approaches to infer information about patterns and processes from high-frequency water quality data to date. For example, Bieroza and Heathwaite^15^ successfully used a fuzzy logic system to determine the direction of the storm event *c*-*q* patterns based on a learning data set including volume of flow discharge and mean air temperature during storm events. Machine learning approaches such as Random Forest or Support Vector Machine tend to outcompete established statistical regression methods, e.g., multiple or partial least-squares regression, in predicting low concentrations of solutes and particulates from high-frequency sensor data.^170^ They are less affected by typical high-frequency data features, e.g., skewed distributions, nonlinear relationships, and multicollinearity,^171^ and can help to establish proxy relationships for solutes for which the appropriate sensor technique is not available.^172^ Thus, machine learning techniques can provide opportunities to establish fully automated high-frequency data control and analysis frameworks, which can be particularly appealing to regulators and stakeholders interested in incorporating high-frequency sampling into existing environmental programs.

High-frequency water quality data offer opportunities to improve conceptual understanding of complex catchments and their role in controlling solute/particulate transport. For example, naturally occurring conservative tracers, such as stable water isotopes and chloride, have been widely used to estimate the transit time of water’s journey through catchments, on its way from rainfall to streamflow. The recent availability of high-frequency tracer time series has revealed catchment transport behavior on time scales comparable to those of catchment hydrological response, thus illuminating new aspects of catchment processes and spurring the development of new analysis tools, including storage age selection approaches (see, e.g., refs (16, 174) ) and ensemble hydrograph separation,^175^ which quantifies streamflow’s average fraction of new water (e.g., same-day precipitation if sampling is daily) as well as its transit time distribution. This latter approach can provide data-driven, model-independent estimates of how catchment transport processes respond to variations in antecedent wetness and precipitation intensity.^19^

Many existing process-based hydrochemical models (e.g., the Soil and Water Assessment Tool [SWAT], Integrated Calibration and Application Tool [INCA]) have been developed in the era of low-frequency data and low computational power, which inhibits straightforward integration of high-frequency data sets. To date, applications have been based on aggregated high-frequency data (e.g., to daily mean concentrations) to match the time step of the model,^176^ or high-frequency data have been used for setting robust model evaluation criteria.^21^ Evaluation of high-frequency data can provide insights into overall model performance and its ability to represent critical transport or turnover processes (e.g., subsurface delivery of solutes) and thus help in selection of appropriate hydrochemical models for a given catchment. Piniewski et al.^177^ showed that using high-frequency data to calibrate a physically based model (SWAT) can improve its performance and that high-frequency data enables benchmarking model predictions and assessing sources of uncertainty in the calibration data. High-frequency chemical data can also help validate model performance by simulating short-term changes in stream concentrations that reflect catchment-specific changes in runoff partitioning and event-based concentration or dilution effects^178^ or hysteretic patterns.^179^

New opportunities for developing pattern detection and modeling approaches for high-frequency data sets could potentially utilize freely available software, where models are provided as services such as the Mobius model builder,^180^ the Cloud Services Innovation Platform,^181^ and the streamPULSE platform.^26^ The Mobius model builder is a freely available tool using a modular approach which implements water quality models such as the INCA and Simply models.^182^ The Cloud Services Innovation Platform is a web interface compatible with a variety of models, requiring no in-house maintenance and with adequate data security tools. The streamPULSE platform facilitates stream metabolism modeling through providing consistent approaches to sensor data collection and protocols for data quality assurance and control and stream metabolism modeling.^26^ Additionally, several freely available toolboxes designed to analyze high-frequency water data have been released in the past years, including the R packages *oddwater* developed to detect outliers in WQ data from in situ sensors,^183^*waterData* which calculates and plots anomalies, ensemble hydrograph separation scripts,^175^ and *EndSplit* for end-member splitting analysis^184^ and Python packages *AbspectroscoPY* to analyze UV–vis sensor data^185^ and *pyhydroqc* for automating detection and correction of anomalies in sensor data.^169^

## 4 Future Directions and Challenges

Kirchner et al.^11^ conceived a vision of a major revolution in catchment science enabled by high-frequency water quality measurements. Now 20 years on, some of these advances have materialized, e.g., clearer quantification of coupling between hydrological and chemical dynamics or better understanding how flow path routing contributes to activation of different solute and particulate stores leading to different water quality responses during storm events. Other advances, such as using high-frequency chemical measurements to test existing hydrological and hydrochemical models, have been less evident, indicating that there is a continuing need to develop the next generation of catchment models that operate on time steps of high-frequency data to fully utilize the wealth of information present in these data sets. Most likely, this progress has been hindered by the great variation in high-frequency water quality patterns among different catchments and solutes/particulates. Thus, to fully realize the potential of high-frequency water quality observations, there is a need to synthesize observed hydrochemical patterns into catchment and solute/particulate typologies that can be the foundation for future conceptual and process-based catchment models.

While high-frequency data sets undoubtedly come with great potential, we do not advocate more widespread high-frequency monitoring purely for the sake of accumulating data, but rather to apply it in situations where conventional water quality monitoring is insufficient to answer key scientific questions. By identifying the future challenges of high-frequency monitoring, we aim to outline strategies for cooperation among institutions and across research disciplines. These challenges should serve as an inspiration for researchers and policymakers to rethink the potential of high-frequency data, to develop new questions, to join forces, and to make the most of the ever-expanding potential of high-frequency water quality data.

As national high-frequency monitoring programs have started to emerge in Europe and North America, emphasis should be put on merging already existing water quality data sets in these regions. However, high-frequency monitoring is largely underrepresented in tropical and cold regions,^186^ which calls for intensified efforts to expand monitoring to enable biome- or continent-wide comparisons. To understand internal catchment processes, we also stress that multiple station measurements and nested sampling strategies are necessary for inferring reach-scale water quality patterns and their stream network evolution.

A future challenge for catchment scientists will be the development of robust high-frequency proxies for existing and emerging environmental contaminants (e.g., antibiotics, microplastics, pesticides), a vast group of constituents that calls for novel sensor technology development. There is a growing body of research establishing quantitative relationships using high-frequency data, e.g., employing machine learning tools, but we advocate more process-based approaches that consider biogeochemical interactions between different solutes/particulates. Combined high-frequency data sets of hydrology and biogeochemistry have further potential for bridging the fields of stream biogeochemistry and ecology, where linkages between stream metabolism and consumer food webs are currently underexplored.^187^ There is also a strong need to develop new measurement technologies to address challenges of high-frequency sampling in intermittently dry or frozen streams.

There is a continuous need to develop and maintain high-frequency data sets to maximize the chances of detecting hydrological or chemical trends that are indicative of global change impacts on freshwater systems and that document expected ranges of variability. Here, collaborative efforts should be undertaken to increase the temporal and particularly spatial resolution of records. Long-term high-frequency data sets are currently managed by individual academic institutions, governments, and private sector institutes and are therefore limited by funding availability. Several data sets already extend longer than a decade and thus create opportunities for identifying long-term trends in short-term water quality dynamics (i.e., storm event dynamics or diurnal cycling). There is an emerging need for synthesis of these high-frequency data sets, which often cover overlapping parameters, to illuminate how catchments and global change factors control water quality and aquatic ecosystem functions at regional to global scales. Developing and maintaining high-frequency data repositories, e.g., the Water Quality Portal managed by the U.S. Environmental Protection Agency, to enable access to high-frequency water quality data sets are particularly important steps in this process.

Merging of individual data sets into accessible formats will require interagency cooperation and is likely to bring challenges in data compilation, quality control, and management that require collaborative and potentially interdisciplinary solutions^188^ and appropriate funding sources to support such initiatives. The recent proliferation of high-frequency monitoring has resulted in an abundance of extensive water quality data sets, which puts the challenge on developing incentives for data sharing (or developing a community culture that demands it), and metadata standards that are straightforward and practical to implement. Here, a balance must be struck between the goals of rigorous quality control and complete documentation versus many research groups’ very limited resources for data curation and dissemination. Additionally, data assurance and quality control standards need to be implemented on a wider basis, especially as new technologies emerge.^10^ With countless statistical and process-based models to choose from, there is a need for the scientific community to be consistent when developing or choosing data analysis models.

Resolving these methodological challenges is also needed to facilitate better integration of high-frequency water quality sampling into regulation and decision making. Realizing the potential of high-frequency data requires synthesis, simplification, and extracting information in ways that are accessible to stakeholders and policymakers. As many environmental agencies and monitoring programs have invested in *in situ* high-frequency monitoring, there is a need for skills and training in data cleaning, quality assurance and control, data wrangling, analysis, and modeling for the “next-generation” high-frequency data collection, e.g., from distributed sensor networks.

Future applications can benefit from coupling high-frequency water quality measurement technology with other novel and high-resolution techniques, including catchment and stream spatial modeling (see, e.g., ref (21)) mass spectrometry (see, e.g., ref (58)), flow field-flow fractionation (see, e.g., ref (189)), biosensors (see, e.g., ref (190)), eddy covariance (see, e.g., ref (191)), acoustic Doppler current profilers [ADCP] (see, e.g., ref (192)), flow-cytometry (see, e.g., ref (193)), drones (see, e.g., ref (194)), passive samplers (see, e.g., ref (195)), remote sensing (see, e.g., ref (196)), telemetry (see, e.g., ref (6)), and citizen science approaches (see, e.g., ref (96)). This fusion of technologies and methods can enable the measurement of a broader range of chemicals and water quality parameters and establishment of direct links between high-temporal and high-spatial resolution sampling and modeling.

High-frequency water quality sensors are part of the greater “high-frequency wave of the present”^10^ in which high-frequency measurements of key elements, e.g., C, N, and P, are becoming readily available for different environments and media such as gases,^197^ soils,^198^ and biological samples with environmental DNA.^199^ Ultimately, using multisensor technologies simultaneously to measure key biogeochemical cycles across Earth subsystems (i.e., atmosphere, soils, waters, biosphere) can lead to new discoveries of elemental and ecosystem interactions and fundamentally improve our estimates of fluxes and turnover rates from river networks.

### 4.1 Concluding Points

High-frequency water quality measurements have generated new insights into the “fine structure of water quality dynamics”.^11^ Measurements at a similar temporal resolution as many hydrological and biogeochemical process rates, previously obscured by low-frequency or sporadic high-frequency sampling, have revolutionized our understanding of catchment and stream processes that shape water quality. As the technology and number of high-frequency sampling data sets evolve further, there is a critical need to synthesize the existing understanding of hydrochemical patterns and process linkages revealed by high-frequency sampling across varied catchments and for different solutes/particulates. High-frequency data sets provide insights into catchment and stream network transport and processing of a range of chemicals, interactions between different biogeochemical cycles and processes, and global change impacts on freshwater systems, thus contributing to advances in catchment science, hydrochemistry, and aquatic ecology. The current challenges are to integrate existing high-frequency data sets for knowledge synthesis to develop protocols for better transparency and data sharing, to expand high-frequency chemical observations in multiple catchments, and to standardize the approaches for quality control, quality assurance, analysis, and modeling of high-frequency data. Standardization of approaches to deal with vast quantities of high-frequency data will be essential for high-frequency water quality measurements to transform from purely scientific endeavors to water quality regulation and decision-making applications. We have yet to discover the full potential of high-frequency water quality technology. Progress will be driven by combining high-frequency water quality instruments with other tools from a kayak to AUVs and remote sensing and state-of-the-art statistical and modeling tools. Thus, we are excited to ride this high-frequency wave of both the present and the future.

## Supporting Information Available

Table S1: Summary of existing and emerging technologies for high-frequency water quality measurements (PDF)
Table S2: Summary of high-frequency hydrochemical data sets available as open access and on request (PDF)
