# Supplementary material for: Advances in Catchment Science, Hydrochemistry, and Aquatic Ecology Enabled by High-Frequency Water Quality Measurements
Source: Environ Sci Technol. 2023 Mar 13;57(12):4701–19. doi: 10.1021/acs.est.2c07798 (PMC10061935; doi:10.1021/acs.est.2c07798)
Supplement: Supplementary file 1 — es2c07798_si_001.pdf [file es2c07798_si_001.pdf]

Table 1 Existing and emerging technologies for high-frequency water quality measurements

TECHNOLOGY	HOW DOES IT WORK	SAMPLING FREQUENCY	ADVANTAGES	LIMITATIONS	EXAMPLE REFERENCES
EXISTING AND ESTABLISHED TECHNOLOGIES					
**Autosamplers**	Stream water is pumped into bottles at specified trigger signals. These are either based on set time intervals or external inputs e.g., certain flow discharge or water level. In most applications up to 24 bottles. Water samples are then analyzed in the laboratory.	From 1 min. Typically, hourly sampling or every 7 hours over a week (24/7 approach)	Easy to set-up. Portable devices allow various sampling modes (grab time- or flow-proportional or composite samples). Low maintenance. Allow for a wide range of parameters measured in the laboratory.	May lack refrigeration, leading to potential sample degradation during long deployments. Limited number of samples. Potential problems with power supply resulting in no samples being collected.	(Audet et al., 2014; Bieroza and Heathwaite, 2016; Jordan and Cassidy, 2011; Neal et al., 2013)
**Electrode-based sensors (amperometric measurements)**	Measurement of electrical current generated during the electrochemical oxidation or reduction of an electro-active species. Allow for *in situ* measurements of temperature, pH, electrical conductivity, dissolved oxygen, redox potential.	From 1 s	Low cost. Low maintenance. Can be used in a multiparameter sondes.	Frequent calibration needed (pH and redox). Require submersion (dissolved oxygen, pH and redox). Sensitive to biofouling.	(Audet et al., 2020; Mahmud et al., 2020; Wade et al., 2012b)

**Ion selective sensors (ISE; potentiometric measurements)**	*In situ* measurement of solute concentration using the electrical potential difference caused by a concentration difference between analytes concentration in solute and the reference material in the electrode. Commonly used for measurements of pH, nitrate, chloride and for ammonium.	From 1 s	Low cost, low power consumption	Frequent calibration needed. Replacement of electrodes and calibration necessary. Prone to drifts and errors at low concentrations. Cross sensitivity between ions (e.g., nitrate and chloride).	(Bende-Michl and Hairsine, 2010; Mahmud et al., 2020)
**UV-VIS absorbance sensors**	*In situ* measurement of the intensity of the UV-VIS light absorbed by the ions which is proportional to their concentration in the solution. Depending on the measured wavelength, measurements of turbidity, total suspended solids, nitrate, dissolved and total organic carbon, biogeochemical oxygen demand, dissolved oxygen, color.	From 1 s	Wide range of available parameters. Easy to set-up and deploy. Low maintenance. Do not require any addition of reagents. Stable measurements which are not sensitive to changes in temperature, pH or salinity.	Precision and detection range depend on optical path length. Not a direct measurement and requires establishing a local calibration curve against grab samples analyzed in the laboratory. Sensitive to drying. Prone to biofouling and signal quenching.	(Ruhala and Zarnetske, 2017; Torres and Bertrand-Krajewski, 2008; Vaughan et al., 2017)
**Fluorescence optical sensors**	*In situ* measurements of the UV-VIS light emitted by organic solutes and particulates present in water. Measured parameters include different fractions of dissolved organic matter, typically peak C (CDOM) and peak T fluorescence, chlorophyll.	From 1 s	Easy to set-up. Low maintenance	Require site-specific correction for signal quenching by temperature, pH, turbidity, metals etc.	(Khamis et al., 2018; Ruhala and Zarnetske, 2017; Saraceno et al., 2009)

**Wet-chemistry analysers**	Stream water is pumped into streambank analysers, which perform laboratory colorimetric, chromatographic, MP-AES and spectroscopic isotope measurements. Measured parameters include nutrients (total phosphorus, total reactive phosphorus, nitrate nitrogen, ammonium nitrogen) and total organic carbon, stable isotopes.	From 10 minutes	Direct and accurate measurement. Controlled analysis conditions (temperature).	Lack of in-built filtration. High maintenance and running cost.	(Bieroza et al., 2014; Jordan et al., 2012; von Freyberg et al., 2022; Wade et al., 2012a)
**EMERGING TECHNOLOGIES**					
**Lab-on-a-chip**	Use microfluids and nanotechnology to measure solutes using colorimetric methods.	From 10 minutes	Low sample volume and potentially low-cost.	Not yet commercially available. Poor signal-to-noise ratio.	(Beaton et al., 2012)
**Nano sensors**	Use nanomaterials with nanostructures that provide high surface-area-to-volume and binding affinity to target molecules using optical, electrochemical, magnetic, or mechanical methods. Measured parameters include pharmaceutical contaminants, pesticides, herbicides, metals, and ions.	Sub hourly	Low detection limits. High selectivity and sensitivity. Potential to measure emerging contaminants.	Not yet commercially available.	(Hairom et al., 2021)

**DNA-based biosensors (genetically encoded)**	Molecular systems that detect and respond to specific targets and are constructed from two modular parts—a sensor and a reporter. First, the sensor recognizes a target of interest and changes its shape to start production of the reporter. The reporter then creates a detectable output using colorimetric or optical methods.	Sub hourly	Detection of arsenic, fluoride, pathogens, and emerging contaminants (e.g., herbicides such as atrazine). Potentially low cost.	No distinction between dead or alive cells. Parts of the sensors are biological, thus lacking validation of field-deployability.	(Thavarajah et al., 2020)
**Molecular biosensors**	Bacterially derived sensors employed to determine the presence, concentration and the bioavailability of both specific chemicals and the overall toxic effect of e.g., heavy metals.	Sub hourly	Detection of low concentrations of a toxic contaminant, with sensitivity over several orders of magnitude.	No portable instrument available.	(Justino et al., 2017; Rampley et al., 2020)
**eDNA portable measurements**	Based on isolation of eDNA from filtered stream water and subsequent amplification and quantification of target genes.	Sub hourly	Detection of biodiversity patterns in steams.	Not commercially available. Markers are needed for each target species.	(Ruppert et al., 2019)
**Flow – cytometry measurements**	Single cell/particle measurement technology that identifies the number of bacterial cells by coloring with fluorescence dye and counting, further discriminations e.g., certain strains, permeability of cell-membrane are also possible.	Sub hourly	Allows insights in dynamics of bacterial contamination, discrimination of phytoplankton abundance.	Currently lack of portable and miniaturized instrument. Requires reagent and generates waste. High maintenance. High cost.	(Pomati et al., 2011)

**Acoustic turbidity measurement**	The measurement method is based on the incoherent backscattering along a beam from an emitted pulse on particles in stream water.	From 1 s	Surrogate signal to measure sediment concentrations. Information on particle size distribution.	Limited examples of stream deployments. Depending on transducer, the range of particle sizes is limited. Prone to errors caused by water turbulence.	(Sirabahenda et al., 2019)
**Fast repetition rate fluorimetry**	An optical technique for estimating photosynthetic properties of phytoplankton from measurements of *in situ* fluorescence.	Sub hourly	Proxy for gross primary production.	Limited examples of stream deployments. Limitations as in fluorescence sensors.	(Zananski et al., 2010)

Audet J, Bastviken D, Bundschuh M, Buffam I, Feckler A, Klemedtsson L, et al. Forest streams are important sources for nitrous oxide emissions. Glob Chang Biol 2020; 26: 629-641.

Audet J, Martinsen L, Hasler B, de Jonge H, Karydi E, Ovesen NB, et al. Comparison of sampling methodologies for nutrient monitoring in streams: uncertainties, costs and implications for mitigation. Hydrology and Earth System Sciences 2014; 18: 4721-4731.

Beaton AD, Cardwell CL, Thomas RS, Sieben VJ, Legiret FE, Waugh EM, et al. Lab-on-chip measurement of nitrate and nitrite for in situ analysis of natural waters. Environ Sci Technol 2012; 46: 9548-56.

Bende-Michl U, Hairsine PB. A systematic approach to choosing an automated nutrient analyser for river monitoring. J Environ Monit 2010; 12: 127-34.

Bieroza MZ, Heathwaite AL. Unravelling organic matter and nutrient biogeochemistry in groundwater-fed rivers under baseflow conditions: Uncertainty in in situ high-frequency analysis. Science of The Total Environment 2016; 572: 1520-1533.

Bieroza MZ, Heathwaite AL, Mullinger NJ, Keenan PO. Understanding nutrient biogeochemistry in agricultural catchments: the challenge of appropriate monitoring frequencies. Environ Sci Process Impacts 2014; 16: 1676-91.

Hairom NHH, Soon CF, Mohamed RMSR, Morsin M, Zainal N, Nayan N, et al. A review of nanotechnological applications to detect and control surface water pollution. Environmental Technology & Innovation 2021; 24.

Jordan P, Cassidy R. Technical Note: Assessing a 24/7 solution for monitoring water quality loads in small river catchments. Hydrology and Earth System Sciences 2011; 15: 3093-3100.

Jordan P, Melland AR, Mellander PE, Shortle G, Wall D. The seasonality of phosphorus transfers from land to water: implications for trophic impacts and policy evaluation. Sci Total Environ 2012; 434: 101-9.

Justino CIL, Duarte AC, Rocha-Santos TAP. Recent Progress in Biosensors for Environmental Monitoring: A Review. Sensors (Basel) 2017; 17.

Khamis K, Bradley C, Hannah DM. Understanding dissolved organic matter dynamics in urban catchments: insights from in situ fluorescence sensor technology. WIREs Water 2018; 5.

Mahmud MAP, Ejeian F, Azadi S, Myers M, Pejcic B, Abbassi R, et al. Recent progress in sensing nitrate, nitrite, phosphate, and ammonium in aquatic environment. Chemosphere 2020; 259.

Neal C, Kirchner J, Reynolds B. Plynlimon research catchment hydrochemistry. NERC Environmental Information Data Centre, 2013.

Pomati F, Jokela J, Simona M, Veronesi M, Ibelings BW. An automated platform for phytoplankton ecology and aquatic ecosystem monitoring. Environ Sci Technol 2011; 45: 9658-65.

Rampley CPN, Whitehead PG, Softley L, Hossain MA, Jin L, David J, et al. River toxicity assessment using molecular biosensors: Heavy metal contamination in the Turag-Balu-Buriganga river systems, Dhaka, Bangladesh. Sci Total Environ 2020; 703: 134760.

Ruhala SS, Zarnetske JP. Using in-situ optical sensors to study dissolved organic carbon dynamics of streams and watersheds: A review. Sci Total Environ 2017; 575: 713-723.

Ruppert KM, Kline RJ, Rahman MS. Past, present, and future perspectives of environmental DNA (eDNA) metabarcoding: A systematic review in methods, monitoring, and applications of global eDNA. Global Ecology and Conservation 2019; 17.

Saraceno JF, Pellerin BA, Downing BD, Boss E, Bachand PAM, Bergamaschi BA. High-frequency in situ optical measurements during a storm event: Assessing relationships between dissolved organic matter, sediment concentrations, and hydrologic processes. Journal of Geophysical Research 2009; 114: G00F09.

Sirabahenda Z, St-Hilaire A, Courtenay SC, van den Heuvel MR. Comparison of Acoustic to Optical Backscatter Continuous Measurements of Suspended Sediment Concentrations and Their Characterization in an Agriculturally Impacted River. Water 2019; 11.

Thavarajah W, Verosloff MS, Jung JK, Alam KK, Miller JD, Jewett MC, et al. A Primer on Emerging Field-Deployable Synthetic Biology Tools for Global Water Quality Monitoring. NPJ Clean Water 2020; 3.

Torres A, Bertrand-Krajewski JL. Partial Least Squares local calibration of a UV-visible spectrometer used for in situ measurements of COD and TSS concentrations in urban drainage systems. Water Sci Technol 2008; 57: 581-8.

Vaughan MCH, Bowden WB, Shanley JB, Vermilyea A, Sleeper R, Gold AJ, et al. High-frequency dissolved organic carbon and nitrate measurements reveal differences in storm hysteresis and loading in relation to land cover and seasonality. Water Resources Research 2017; 53: 5345-5363.

von Freyberg J, Rucker A, Zappa M, Schlumpf A, Studer B, Kirchner JW. Four years of daily stable water isotope data in stream water and precipitation from three Swiss catchments. Sci Data 2022; 9: 46.

Wade AJ, Palmer-Felgate EJ, Halliday SJ, Skeffington RA, Loewenthal M, Jarvie HP, et al. From existing in situ, high-resolution measurement technologies to lab-on-a-chip – the future of water quality monitoring? Hydrology and Earth System Sciences Discussions 2012a; 9: 6457-6506.

Wade AJ, Palmer-Felgate EJ, Halliday SJ, Skeffington RA, Loewenthal M, Jarvie HP, et al. Hydrochemical processes in lowland rivers: insights from in situ, high-resolution monitoring. Hydrology and Earth System Sciences 2012b; 16: 4323-4342.

Zananski TJ, Twiss MR, Mihuc TB. Use of fluorimetry to evaluate atrazine toxicity to phytoplankton communities. Aquatic Ecosystem Health & Management 2010; 13: 56-65.
