# Supplementary material for: Advances in Catchment Science, Hydrochemistry, and Aquatic Ecology Enabled by High-Frequency Water Quality Measurements
Source: Environ Sci Technol. 2023 Mar 13;57(12):4701–19. doi: 10.1021/acs.est.2c07798 (PMC10061935; doi:10.1021/acs.est.2c07798)
Supplement: Supplementary file 2 — es2c07798_si_002.pdf [file es2c07798_si_002.pdf]

Table 2 High-frequency hydrochemical datasets available as open access and on request

Dataset	Description	High-frequency data	Data availability	References
Plynlimon catchments (UK)	This dataset includes rainfall, river and stream hydro-chemistry data from the River Hafren (Severn)	50 chemical determinands sampled on a weekly basis (1983-2008), over 4 years (2005-2009) of weekly water isotopes, 2 years (2007-2009) of 7-hourly chemical and discharge data	Open access https://catalogue.ceh.ac.uk/documents/551a10ae-b8ed-4ebd-ab38-033dd597a374<br><br>(Kirchner J.W. et al., 2019)	(Kirchner and Neal, 2013; Knapp et al., 2019; Neal et al., 2013a; Neal et al., 2013b)
NEON (the National Ecological Observatory Network; US)	81 freshwater and terrestrial field sites, across 20 ecoclimatic domains	15 min data on chlorophyll a, FDOM, nitrate nitrogen, pH, specific conductivity temperature, turbidity, discharge (various timespans, 2016-)	Open access<br><br>https://data.neonscience.org/	(Edmonds et al., 2022; Hensley et al., 2021)
Demonstration Test Catchments (UK)<br><br>https://catchmentbasedapproach.org/learn/demonstration-test-catchments-dtc/	Three catchments (Eden, Wensum, Avon) with data evidencing how diffuse pollution from agriculture can controlled to improve and maintain water quality	Sub-hourly (15 and 30 min) data on dissolved oxygen, nitrate nitrogen, total phosphorus, total reactive phosphorus, turbidity, discharge (various timespans, 2011-)	Open access<br><br>http://www.environmentdata.org/	(Cooper et al., 2020)
Iowa water quality (US)	30 sampling sites monitored by IHR—Hydroscience & Engineering and USGS with access to real time nutrient data with a range of water-related information	15 min data on dissolved oxygen, nitrate nitrogen, pH, specific conductivity, temperature, turbidity, discharge (various timespans, 2015-)	Open access<br><br>https://iwqis.iowawis.org/	-
United States Geological Survey (USGS) water data	Over 13000 real-time stream, lake, reservoir meteorological,	15 min data on chlorophyll fluorescence, dissolved oxygen, nitrate nitrogen, pH, specific	Open access<br><br>https://dashboard.waterdata.usgs.gov/<br>https://waterdata.usgs.gov/nwis	(Vaughan et al., 2019)

	hydrological and water quality data.	conductivity, temperature, turbidity, discharge (various timespans, 2012-)		
OZCAR datasets (Observatoires de la Zone Critique: Applications et Recherche; France)<br><br>https://www.ozcar-ri.org/fr/ozcar-3/	Twenty-one research observatories and more than 60 instrumented sites ranging from plots to the largest river basins in the world	Basic water quality parameters readily available (pH, redox potential, specific conductivity, temperature, turbidity). Solute concentration data undergo calibration/validation thus access may be limited	Open access/on request https://deims.org/<br><br>Contact individual site for data access	(Aubert et al., 2014; Benettin et al., 2020; Legout et al., 2021)
Oyster River, Cart Creek, Parker River, Ipswich River (US)	A number of catchments of various sizes and land uses. Nested catchments	15 min data on dissolved oxygen, nitrate nitrogen, specific conductivity, temperature, discharge (various timespans, 2013-)	Open access/on request Wilfred Wollheim Wil.wollheim@unh.edu<br><br>https://pie-lter.ecosystems.mbl.edu/data?keys=&field_core_areas_tid=All&field_station_keywords_ter_tid=768	(Wollheim et al., 2017)
Krycklan catchments (Sweden)<br><br>https://www.slu.se/en/departments/field-based-forest-research/experimental-forests/vindeln-experimental-forests/krycklan/infrastructure/	Krycklan is a 67.8 km^2 catchment composed of a mosaic of wetlands and lakes in the boreal forest landscape. Nested catchments	15 min data on dissolved organic carbon, nitrate nitrogen, specific conductivity, temperature, discharge (various timespans, 2016-)	Open access/on request Hjalmar Laudon hjalmar.laudon@slu.se<br><br>https://data.krycklan.se/	(Zhu et al., 2021)
River Leith catchment (UK)	Mesoscale catchment (54 km^2, third Strahler order), clay loam and silty loam soils, agricultural land use (85% permanent grassland). Intensive surface – groundwater interactions	Hourly data on dissolved oxygen, nitrate nitrogen, pH, redox potential, temperature, total phosphorus, total reactive phosphorus, turbidity, discharge (2009-2014)	Open access/on request Magdalena Bieroza magdalena.bieroza@slu.se and Louise Heathwaite louise.heathwaite@lancaster.ac.uk	(Bieroza and Heathwaite, 2015; Bieroza et al., 2014; Heathwaite and Bieroza, 2021)

Hestadbäcken catchment (Sweden)	Headwater catchment (7.4 km^2), clay soils, arable (54%) land use. Mitigation measures for reducing diffuse nutrient losses and erosion and longitudinal turbidity measurements	Low-frequency hydrochemical data (1989-).<br>15 min data on dissolved organic carbon, nitrate nitrogen, temperature, total phosphorus, total reactive phosphorus, turbidity, discharge (2017-)	Open access/on request Magdalena Bieroza magdalena.bieroza@slu.se	(Bieroza et al., 2019; Bieroza et al., 2018)
TERENO (TERrestrial ENvironmental Observatories, Germany)<br><br>https://www.tereno.net/	A number of catchment and monitoring sites of various sizes and land uses. Nested catchments	15 min data on nitrate nitrogen, pH, redox potential, specific conductivity, temperature, turbidity, discharge (various timespans, 2012-)	Open access/on request<br><br>https://ddp.tereno.net/ddp/	(Musolff et al., 2021; Rode et al., 2016; Winter et al., 2021a; Winter et al., 2021b)
Alptal (Switzerland) daily stable isotope time series	Mesoscale alpine catchment (47 km^2) and two sub catchments (0.7 and 1.6 km^2)	30 min data on stable isotopes (deuterium and oxygen-18) and major ions, 5 min data on specific conductivity (2016-)	Open access/on request<br><br>https://www.wsl.ch/en/about-wsl/instrumented-field-sites-and-laboratories/experimented-field-sites-for-natural-hazards/torrent-investigation-in-the-alptal/site-description.html	(Knapp et al., 2020; von Freyberg et al., 2022; von Freyberg et al., 2017; von Freyberg et al., 2018)
BES LTER project (US)<br><br>http://beslter.org	A number of catchments with varied sizes and land uses (urban, forested, agricultural). Nested catchments	15 min data on dissolved organic carbon, nitrate nitrogen, turbidity, discharge (various timespan from 2017-)	On request Jonathan Duncan jxd523@psu.edu	(Duncan et al., 2017)
Agricultural Catchments Programme (Ireland)<br><br>https://www.teagasc.ie/environment/water-quality/agricultural-catchments/	Six catchments covering a range of landscape, soil, farming conditions	15 min data on nitrate nitrogen, specific conductivity, total phosphorus, total reactive phosphorus, turbidity, discharge (2010-)	On request Per-Erik Mellander	(Mellander and Jordan, 2021; Shore et al., 2017; Vero et al., 2019)

HydroShare	HydroShare is a collaborative environment for sharing hydrologic data and models for hydrologists to address critical water issues. Various catchments.	Various determinands and various time spans	Open access<br><br>https://www.hydroshare.org/home/	(Musolff et al., 2021; Winter et al., 2021b)

Aubert AH, Kirchner JW, Gascuel-Odoux C, Faucheux M, Gruau G, Merot P. Fractal water quality fluctuations spanning the periodic table in an intensively farmed watershed. Environ Sci Technol 2014; 48: 930-7.

Benettin P, Fovet O, Li L. Nitrate removal and young stream water fractions at the catchment scale. Hydrological Processes 2020; 34: 2725-2738.

Bieroza M, Bergström L, Ulén B, Djodjic F, Tonderski K, Heeb A, et al. Hydrologic Extremes and Legacy Sources Can Override Efforts to Mitigate Nutrient and Sediment Losses at the Catchment Scale. Journal of Environmental Quality 2019; 48: 1314-1324.

Bieroza MZ, Heathwaite AL. Seasonal variation in phosphorus concentration–discharge hysteresis inferred from high-frequency in situ monitoring. Journal of Hydrology 2015; 524: 333-347.

Bieroza MZ, Heathwaite AL, Bechmann M, Kyllmar K, Jordan P. The concentration-discharge slope as a tool for water quality management. Sci Total Environ 2018; 630: 738-749.

Bieroza MZ, Heathwaite AL, Mullinger NJ, Keenan PO. Understanding nutrient biogeochemistry in agricultural catchments: the challenge of appropriate monitoring frequencies. Environ. Sci.: Processes Impacts 2014; 16: 1676-1691.

Cooper RJ, Hiscock KM, Lovett AA, Dugdale SJ, Sunnenberg G, Vrain E. Temporal hydrochemical dynamics of the River Wensum, UK: Observations from long-term high-resolution monitoring (2011-2018). Sci Total Environ 2020; 724: 138253.

Duncan JM, Welty C, Kemper JT, Groffman PM, Band LE. Dynamics of nitrate concentration-discharge patterns in an urban watershed. Water Resources Research 2017; 53: 7349-7365.

Edmonds JW, King KBS, Neely MB, Hensley RT, Goodman KJ, Cawley KM. Using large, open datasets to understand spatial and temporal patterns in lotic ecosystems: NEON case studies. Ecosphere 2022; 13.

Heathwaite AL, Bieroza M. Fingerprinting hydrological and biogeochemical drivers of freshwater quality. Hydrological Processes 2021; 35: e13973.

Hensley R, Harrison N, Goodman K, Cawley K, Litt G, Nance B, et al. A comparison of water quality sensor deployment designs in wadeable streams. Limnology and Oceanography: Methods 2021; 19: 673-681.

Kirchner J.W., Knapp J.L.A., Schlumpf A, Neal C, Neal M. Stable water isotopes in precipitation and streamflow at Plynlimon, Wales, UK, EnviDat, 2019.

Kirchner JW, Neal C. Universal fractal scaling in stream chemistry and its implications for solute transport and water quality trend detection. Proc Natl Acad Sci U S A 2013; 110: 12213-8.

Knapp JL, Freyberg Jv, Studer B, Kiewiet L, Kirchner JW. Concentration-discharge relationships vary among hydrological events, reflecting differences in event characteristics. Hydrology and Earth System Sciences Discussions 2020: 1-27.

Knapp JLA, Neal C, Schlumpf A, Neal M, Kirchner JW. New water fractions and transit time distributions at Plynlimon, Wales, estimated from stable water isotopes in precipitation and streamflow. Hydrology and Earth System Sciences 2019; 23: 4367-4388.

Legout C, Freche G, Biron R, Esteves M, Navratil O, Nord G, et al. A critical zone observatory dedicated to suspended sediment transport: The meso-scale Galabre catchment (southern French Alps). Hydrological Processes 2021; 35.

Mellander P-E, Jordan P. Charting a perfect storm of water quality pressures. Science of The Total Environment 2021; 787: 147576.

Musolff A, Zhan Q, Dupas R, Minaudo C, Fleckenstein JH, Rode M, et al. Spatial and Temporal Variability in Concentration-Discharge Relationships at the Event Scale. Water Resources Research 2021; 57: e2020WR029442.

Neal C, Kirchner J, Reynolds B. Plynlimon research catchment hydrochemistry. NERC Environmental Information Data Centre, 2013a.

Neal C, Reynolds B, Kirchner JW, Rowland P, Norris D, Sleep D, et al. High-frequency precipitation and stream water quality time series from Plynlimon, Wales: an openly accessible data resource spanning the periodic table. Hydrological Processes 2013b; 27: 2531-2539.

Rode M, Halbedel Nee Angelstein S, Anis MR, Borchardt D, Weitere M. Continuous In-Stream Assimilatory Nitrate Uptake from High-Frequency Sensor Measurements. Environ Sci Technol 2016; 50: 5685-94.

Shore M, Murphy S, Mellander P-E, Shortle G, Melland AR, Crockford L, et al. Influence of stormflow and baseflow phosphorus pressures on stream ecology in agricultural catchments. Science of The Total Environment 2017; 590-591: 469-483.

Vaughan MCH, Bowden WB, Shanley JB, Vermilyea A, Schroth AW. Shining light on the storm: in-stream optics reveal hysteresis of dissolved organic matter character. Biogeochemistry 2019; 143: 275-291.

Vero SE, Daly K, McDonald NT, Leach S, Sherriff SC, Mellander P-E. Sources and Mechanisms of Low-Flow River Phosphorus Elevations: A Repeated Synoptic Survey Approach. Water 2019; 11.

Winter C, Lutz SR, Musolff A, Kumar R, Weber M, Fleckenstein JH. Disentangling the impact of catchment heterogeneity on nitrate export dynamics from event to long-term time scales. Water Resources Research 2021a; 57: e2020WR027992.

Winter C, Tarasova L, Lutz SR, Musolff A, Kumar R, Fleckenstein J. Explaining the Variability in High-Frequency Nitrate Export Patterns Using Long-Term Hydrological Event Classification. Water Resources Research 2021b; 58.

Wollheim WM, Mulukutla GK, Cook C, Carey RO. Aquatic Nitrate Retention at River Network Scales Across Flow Conditions Determined Using Nested In Situ Sensors. Water Resources Research 2017; 53: 9740-9756.

von Freyberg J, Rucker A, Zappa M, Schlumpf A, Studer B, Kirchner JW. Four years of daily stable water isotope data in stream water and precipitation from three Swiss catchments. Sci Data 2022; 9: 46.

von Freyberg J, Studer B, Kirchner JW. A lab in the field: high-frequency analysis of water quality and stable isotopes in stream water and precipitation. Hydrology and Earth System Sciences 2017; 21: 1721-1739.

von Freyberg J, Studer B, Rinderer M, Kirchner JW. Studying catchment storm response using event- and pre-event-water volumes as fractions of precipitation rather than discharge. Hydrology and Earth System Sciences 2018; 22: 5847-5865.

Zhu X, Chen L, Pumpanen J, Keinanen M, Laudon H, Ojala A, et al. Assessment of a portable UV-Vis spectrophotometer's performance for stream water DOC and Fe content monitoring in remote areas. Talanta 2021; 224: 121919.
